# Tire–Pavement Contact-Aware Weight Estimation for Multi-Sensor WIM Systems

**DOI:** 10.3390/s19092027

**Published:** 2019-04-30

**Authors:** Zhixin Jia, Kaiya Fu, Mengxiang Lin

**Affiliations:** 1 State Key Laboratory of Software Development Environment, School of Mechanical Engineering & Automation, Beihang University, Beijing 100083, China; jiazx@buaa.edu.cn; 2 School of Mechanical Engineering & Automation, Beihang University, Beijing 100083, China; sy1607504@buaa.edu.cn

**Keywords:** multi-sensor weigh-in-motion system, BP neural network, signal identification, vehicle weight estimation

## Abstract

Accurately estimating the weight of a moving vehicle at normal speed remains a challenging problem due to the complex vehicle dynamics and vehicle–pavement interaction. The weighing technique based on multiple sensors has proven to be an effective approach to this task. To improve the accuracy of weigh-in-motion (WIM) systems, this paper proposes a neural network-based method integrating identification and predication. A backpropagation neural network for signal classification (BPNN-i) was designed to identify ideal samples acquired by load sensors closest to the tire-pavement contact area. After that, ideal samples were used to predict the gross vehicle weight by using another backpropagation neural network (BPNN-e). The dataset for training and evaluation was collected from a multiple-sensor WIM (MS-WIM) system deployed in a public road. In our experiments, 96.89% of samples in the test set had an estimation error of less than 5%.

## 1. Introduction

Overloading has always been a major concern for road traffic [1]. To date, several weighing techniques have been used for overweight vehicle detection [2,3]. Weigh-in-motion (WIM) is a developing technology that aims at obtaining the gross weight of a vehicle travelling at normal speed [4,5]. Compared with static weighing, two major factors should be considered in designing a WIM system. First, sensors in a WIM system measure dynamic load [6,7] rather than the static weight due to vehicle dynamics [8,9]. Second, the complex interaction between vehicles and pavements leads to difficulties in accurately measuring dynamic load [1,10,11,12,13].

These problems have motivated multiple-sensor WIM systems (MS-WIMs) in which an array of sensors installed inside the pavements is used to sense dynamic force varying with a certain frequency applied by a travelling vehicle. In the past few decades, the studies on MS-WIM systems have mainly focused on two aspects: sensor layout and vehicle weight estimation algorithms. The former concerns how to arrange sensors to precisely measure the dynamic response along the travelling direction. The goal of the latter is to precisely compute the vehicle weight that could eliminate the errors caused by the vehicle dynamics as much as possible. The most related work presented recently in the literature is reviewed as follows. M.H. Glover et al. [14] arranged nine strip sensors at regular intervals. The static weight of the vehicle is estimated by the mean value, the median value, or the average of the highest and lowest values obtained by the sensors [15]. David Cebon et al. [16] deployed 96 strip WIM sensors in Indiana and averaged the outputs of all the sensors to estimate the static weight. Piotr Burnoset et al. [17] evenly placed 16 piezoelectric load sensors in the form of 4-m-long strips in Poland. The static weight of a vehicle was computed as a simple average value of the load samples from successive sensors. Wenbin Zhang et al. [18] used five embedded concrete strain sensors, and the static weight was also computed as a simple average value of all the sensors. Sungkon Kim et al. [19] arranged 12 strain sensors in three rows and four columns at equal intervals in Korea and three sensors in one row at equal intervals in the direction perpendicular to the driving. These strain sensors were mounted on the lower surface of the beam of a bridge. An artificial neural network (ANN) was used to fuse the measurements of multiple sensors to estimate the static weight [19,20,21,22]. Ryszard Sroka et al. [23] used 16 polymer piezoelectric load sensors, 8 inductive loop sensors, and 8 temperature sensors in their MS-WIM. The sensors were distributed evenly along the site. The static weight was estimated using a method of cooperative fusion, complementary fusion, and attribute fusion.

Unfortunately, the application of multiple-sensor WIM systems to weight enforcement is still limited due to their poor accuracy [4,24,25]. Multiple factors contribute to this problem [26,27,28]. Research on vehicle and road interaction has revealed that the instantaneous force applied by a moving wheel can be approximated by a Gaussian distribution. The mean value of the Gaussian distribution is approximately linear with the dynamic load applied by the vehicle, and occurs at the centerline of the wheel path [29,30]. Inspired by these studies, we propose a novel gross vehicle weight estimation method for MS-WIM systems. The signals of the sensors closest to the tire—pavement contact area rather than all signals collected by an MS-WIM system are used to compute the gross vehicle weight of a passing vehicle [31]. Specifically, two kinds of backpropagation neural network are designed to classify signals and predict vehicle weight because they allow us to tackle those tasks that are too difficult to solve by analytical methods. The proposed method is implemented for an MS-WIM system deployed on a public road, and the data obtained from real road traffic are used to train and test our identification network and prediction network.

## 2. Layout of the Sensors

**Figure 1:**
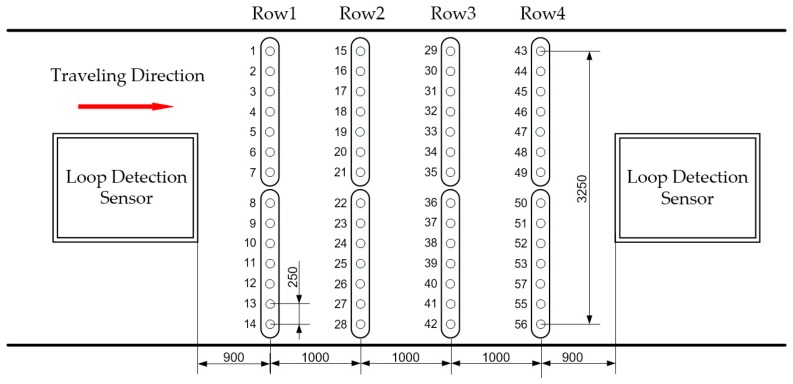
The layout of the sensors.

The layout of the sensors of the MS-WIM system we used is shown in Figure 1. A total of 56 load sensors were arranged in an array of 4 rows and 14 columns. Load sensors were customized strain gauges in a half-bridge configuration with a temperature compensation module. Two loop-detection sensors were placed at both ends of the sensor array. When a vehicle passed through the first loop sensor, the system began to acquire the output of each load sensor, and the signal collection process ended after the vehicle passed through the second loop sensor. At the same time, the average speed of the vehicle was computed, since the distance between the loop sensors was known. The output signal of a load sensor was considered as one sample—that is, 56 samples were collected after a vehicle passed. Seven load sensors in a row were grouped and mounted in a steel box. All steel boxes were embedded 30 cm below the road surface.

## 3. Ideal Sample Identification

**Figure 2:**
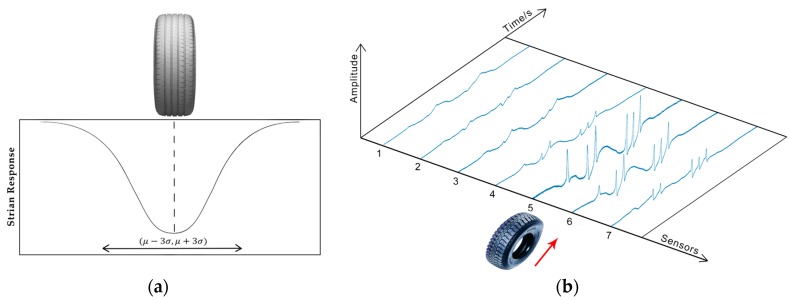
(**a**) Strain response under a moving wheel adapted from [29]; (**b**) An example of samples.

Previous studies on the interaction between moving vehicles and roads have revealed that the dynamic load applied by a moving wheel is approximatively Gaussian [29]. As shown in Figure 2a adapted from [29], the strain just below the tire is the mean of the Gaussian distribution and would decay rapidly due to the mechanical properties of roads. In the case of the MS-WIM system, an array of sensors were deployed to sense dynamic load along the travelling direction. However, not all the sensors in the array could properly detect instantaneous forces applied by a moving wheel. Figure 2b shows an example of output signals obtained from our MS-WIM system for a six-axis vehicle. When the wheels passed over the sensors, the closer the sensors were to the tire—pavement contact area, the stronger the output signals were. For those sensors far away from the wheel, the output signals were too weak to be used. We refer to the output signals generated by the former sensors as ideal samples in this work. For our example in Figure 2b, samples generated from sensors 5 and 6 are ideal.

The values of ideal samples are measurements of tire—pavement contact stress. Most prior work has used ideal samples implicitly to estimate the vehicle/axis static weight by controlling the trajectory of vehicles [16,29]. However, in reality, we are even unable to know the exact locations of the wheels ahead of time for a vehicle travelling on the road. Therefore, a general solution to identify ideal samples is necessary.

### 3.1. Data Preprocessing

The signals that were originally acquired needed to be appropriately processed to facilitate subsequent processing. The preprocess proceeded in two steps: normalization and transformation. Normalization is related to the magnitude of signals, which depends on the dynamic force applied and the characteristics of sensors. To eliminate possible biases, the baseline of the original signal was corrected, and the relative magnitude of signals was derived as per Equation (1):

(1) $$A=\left(A-A_{min}\right)/\left(A_{max}-A_{min}\right),$$

where:

*A*—the value of the data point to be normalized;
$A_{min}$—the minimum value of all data points in the signal; and
$A_{max}$—the maximum value of all data points in the signal.

**Figure 3:**
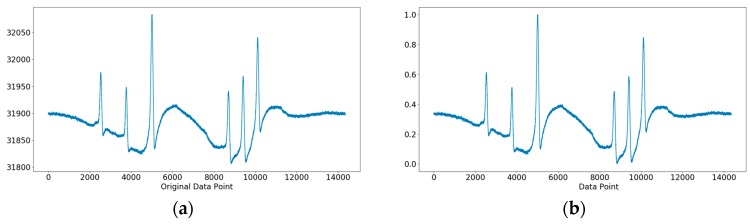
(**a**) The original signal; (**b**) The signal after normalization.

Figure 3 shows an original signal and its results after normalization. All the data points’ values were between 0 and 1, and the relative magnitude and trend of the data points’ values were not changed after normalization, but the differences in the peak sizes of different signals were eliminated.

Moreover, the sampling frequency of an MS-WIM system is usually fixed. This means that the number of data points in the signals varies with vehicle speed. Therefore, the data needed to be transformed so that all signals had the same length. To reduce the impact of the transformation on signals, the target length of a signal was set as the median of the lengths of all signals. In the case that the length of the signal was greater than the target length, the signal was sparsely sampled at a constant interval. The interval is given in Equation (2):

(2) $$I=\left\lfloor L_{ori}/(L_{ori}-L_{tar})\right\rfloor$$

where:

I—the interval;
$L_{ori}$—the original length of the signal;
$L_{tar}$—the target length of the signal; and
⌊ ⌋—the round down operation.

Otherwise, the signal was interpolated by inserting a data point at a constant interval. The interval is given in Equation (3), and the value of the data point to be interpolated is given in Equation (4):

(3) $$I=\left\lfloor L_{ori}/\left(L_{tar}-L_{ori}\right)\right\rfloor$$

(4) $$A_{pos}=\left(A_{pos-1}+A_{pos+1}\right),$$

where:

$A_{pos}$—the value of the data point to be interpolated;
$A_{pos-1}$—the value of the previous data point of the data point to be interpolated; and
$A_{pos+1}$—the value of the next data point of the data point to be interpolated.

**Figure 4:**
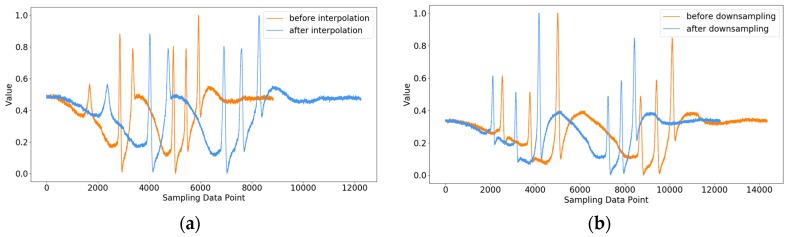
(**a**) The signal before/after interpolation; (**b**) The signals before/after down-sampling.

Figure 4 shows the samples before and after signal transformation, in which the target length of a signal is 12,200.

### 3.2. Short-Time Fourier Transform

The signal generated by a load sensor is a kind of non-stationary time-domain signal [12]. The noise exists during the whole process of data collection, and the effective signal occurs only when the wheel passes over the sensor. In order to obtain the time-domain and frequency-domain characteristics of the effective signal, the short-time Fourier transform was used to extract features for classifying signals.

The basic idea of the short-time Fourier transform is to divide a signal into a number of segments and perform a Fourier analysis segment-by-segment to obtain a local spectrum diagram in different time segments by a window function with an appropriate width to continuously move along the time axis. The definition of the short-time Fourier transform is given in Equation (5):

(5) $$X\left(\tau ,\omega \right)=\int_{-\infty }^{\infty }x\left(t\right)\omega \left(t-\tau \right)e^{-j\omega t}dt,$$

where:

x(t)—the time-varying signal, that is, the signal to be transformed;
t—the time variable;
ω—the angular frequency;
ω(t)—the window function;
τ—the window time position of window function; and
X(τ,ω)—the time-frequency function, which reflects the spectral amplitude of the component in which the frequency is ω of x(t) at time *t*.

For the short-time Fourier transform, the selection of the window function is important because the result of the short-time Fourier transform is mainly determined by the width and shape of the window function. According to the characteristics of the signals, Hamming Window was selected as the window function, as defined in Equation (6):

(6) $$\omega \left(n,\alpha \right)=\left(1-\alpha \right)-\alpha \times \cos\frac{2\pi n}{N-1},$$

where:

α—the scale factor, which is generally 0.46 [32,33];
n—the width of the window function, and 0 ≤ *n* ≤ *N* − 1.

After the short-time Fourier transform, the features of signals, i.e., the local spectrograms of each signal in different time periods, were obtained, which would further be fed into a neural network for classification.

### 3.3. Signal Classification

**Figure 5:**
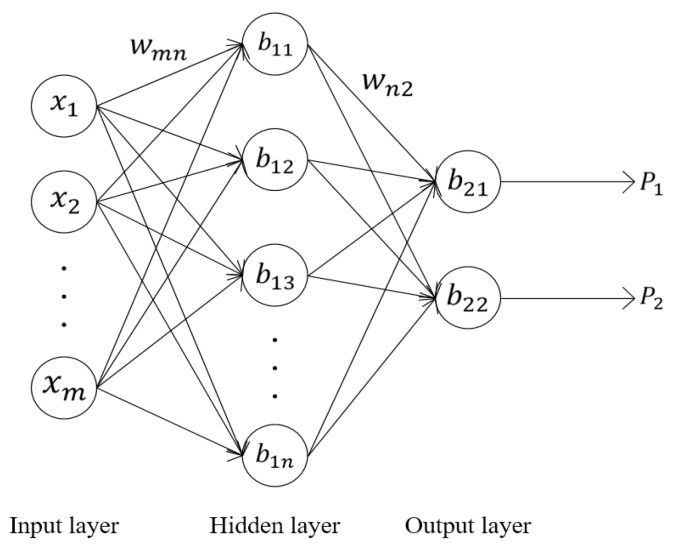
The structure of backpropagation neural network for identification (BPNN-i).

A three-layer forward neural network (BPNN-i) was designed to classify the signals. As shown in Figure 5, *x_i_* (1 ≤ *i* ≤ *m*) are the input features of the BPNN-i, *b_1j_* (1 ≤ *j* ≤ *n*) is the bias values of neurons in the hidden layer, $b_{21}$ and $b_{22}$ are the bias values of the output neurons, $w_{mn}$ is the weights between the input layer and the hidden layer, and $w_{n2}$ is the weights between the hidden layer and the output layer. The number of neurons in the hidden layer was set to 512. The outputs of the output layer neurons were the probability that the sample belongs to each category. The number of neurons in the output layer was the number of categories, that is, two, where one represents an ideal sample and the other is not.

The number of neurons in the input layer of the BPNN-i depended on the dimension of the spectral amplitude of a signal. However, the dimension of the spectral amplitude obtained by the short-time Fourier transform was high due to the high sampling frequency of the MS-WIM system. Principal component analysis (PCA) was used first to reduce the dimension of the signal spectral amplitude into a few irrelevant integrated components.

The activation function of neurons in the hidden layer was set as a sigmoid function, defined as Equation (7):

(7) $$f_{1}\left(x\right)=\frac{1}{1+e^{-x}}.$$

So, the output of the neurons in the hidden layer was:

(8) $$H=f_{1}\left(W_{1}^{T}X+B_{1}\right),$$

where:

$H=\left[h_{1},h_{2},\cdots ,h_{n}\right]$—the output of the hidden layer;
$X=\left[x_{1},x_{2},\cdots ,x_{m}\right]$—the input of BPNN-i, that is, the spectral amplitude after PCA;
$W_{1}=\left[\begin{array}{ccc} w_{11}^{1} & \cdots & w_{1n}^{1}\\ \vdots & \ddots & \vdots \\ w_{m1}^{1} & \cdots & w_{mn}^{1} \end{array}\right]$—the weights between the input layer and the hidden layer;
$B_{1}=\left[b_{11},b_{12},\cdots ,b_{1n}\right]$—the bias values of neurons in the hidden layer.

The activation function of neurons in the output layer was set as a linear function, which is defined as Equation (9):

(9) $$f_{2}\left(x\right)=x.$$

So, the output of BPNN-i is:

(10) $$P=f_{2}\left(W_{2}^{T}H+B_{2}\right),$$

where:

$P=\left[p_{1},p_{2}\right]$—the output of the output layer;
$W_{2}=\left[\begin{array}{cc} w_{11}^{2} & w_{12}^{2}\\ \vdots & \vdots \\ w_{n1}^{2} & w_{n1}^{2} \end{array}\right]$—the weights between the hidden layer and the output layer; and
$B_{2}=\left[b_{21},b_{22}\right]$—the bias values of neurons in the output layer.

## 4. Gross Vehicle Weight Estimation

So far, ideal samples and information about vehicle axles were obtained after a vehicle passed. Our ultimate goal was to estimate vehicle weight by ideal samples. Like the previous signal identification, we relied on BP networks in which features such as crests were network inputs.

### 4.1. Data Preprocess

In order to extract crests in a signal accurately, we had to increase the difference between crests and non-crests so as to reduce the effect of noise. To this end, crest sharpening was applied by Equation (11):

(11) $$y^{\prime }=y-f_{1}\frac{d^{2}y}{dx^{2}}+f_{2}\frac{d^{4}y}{dx^{4}},$$

where:

y—the original signal;
$y^{\prime }$—the processed signal;
$f_{1}$—the scale factor of the second derivative of the original signal; and
$f_{2}$—the scale factor of the fourth derivative of the original signal.

**Figure 6:**
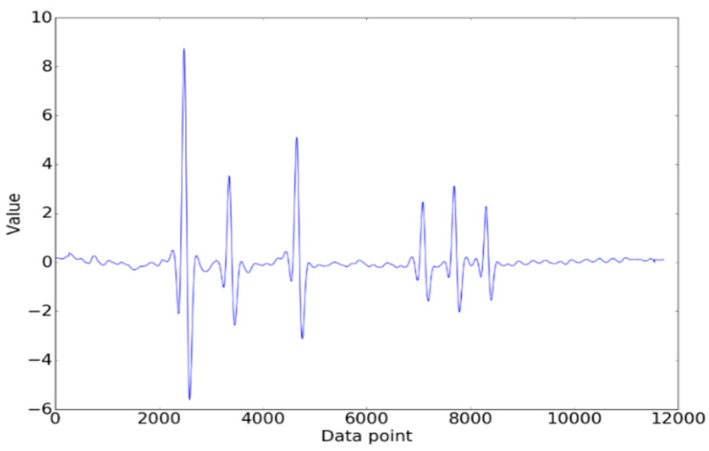
The signal after normalization.

Because the magnitude of the crest was relevant to the weight of the vehicle, the signals generated by vehicles of different weights were quite different. Z-score normalization was applied to reduce this kind of difference. The result is shown in Figure 6.

(12) $$y^{\prime }=\frac{y-\bar{y}}{\sigma },$$

where:

y—the original signal;
$y^{\prime }$—the signal after normalization;
$\bar{y}$—the average of the original signals; and
σ—the standard error of the original signals.

### 4.2. Extraction of Crest

When the wheel of a vehicle passes over a sensor, contact stress causes an obvious fluctuation in the sensor output. To locate the signal wave crest, a local search algorithm was used. Specifically, we referred to the trough point on the left side of the crest as the *contact point*, while the trough points on the right side of the crest were referred to as the *leaving point*, which indicate the positions where the wheel starts pressing the sensor and leaves the sensor, respectively. The signal between the contact point and the leaving point is the crest to be extracted.

**Figure 7:**
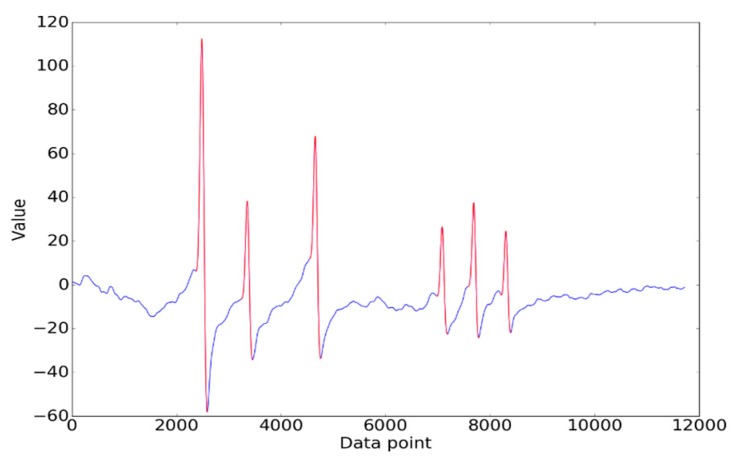
The ideal result of crest extraction.

**Figure 8:**
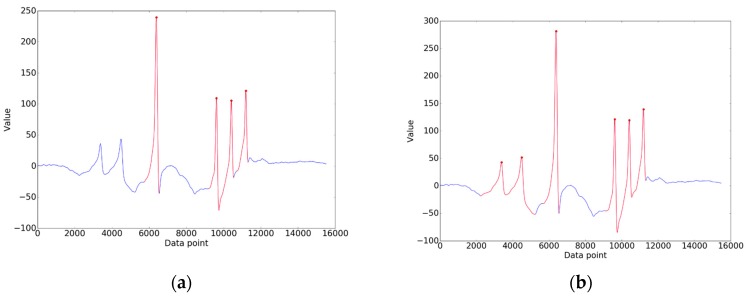
(**a**) The result with missed crests; (**b**) The result after crest correction.

In the ideal situation, the extraction result is shown in Figure 7. That is, the number of crests extracted was equal to the number of axles of the vehicle. However, as shown in Figure 8a, some crests could have been missed due to complicated circumstances during sampling. Crest extraction correction was therefore introduced. Firstly, the number of axles was determined according to the number of crests occurring mostly for all signals collected. The signals whose number of crests extracted was equal to the number of axles were regarded as reference signals, and others were signals to be corrected. Secondly, the missing crests in a signal were further complemented by matching crests with the reference signal. The result after crest matching is shown in Figure 8b.

### 4.3. Estimation of Vehicle Weight

**Figure 9:**
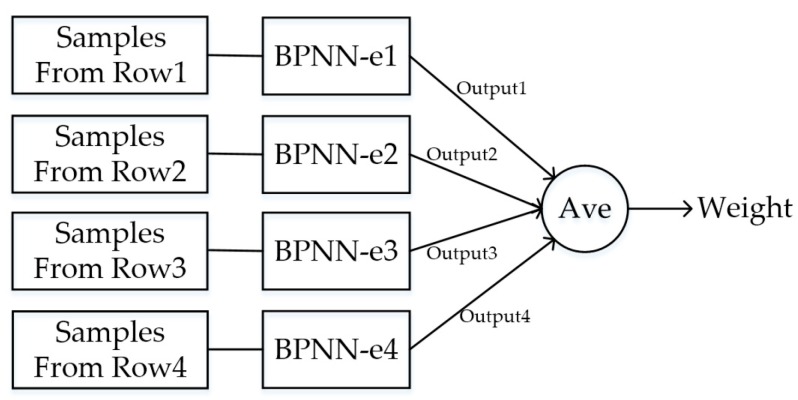
Weight calculation on the neural networks for estimation (BPNN-e).

A multi-layer forward neural network (BPNN-e) was designed to compute the gross weights of vehicles using data collected by the sensors of each row in the WIM system. Therefore, as shown in Figure 9, there were four BPNN-es corresponding to four rows of sensors, and the weight of a vehicle was the average of the outputs of all BPNN-es.

**Figure 10:**
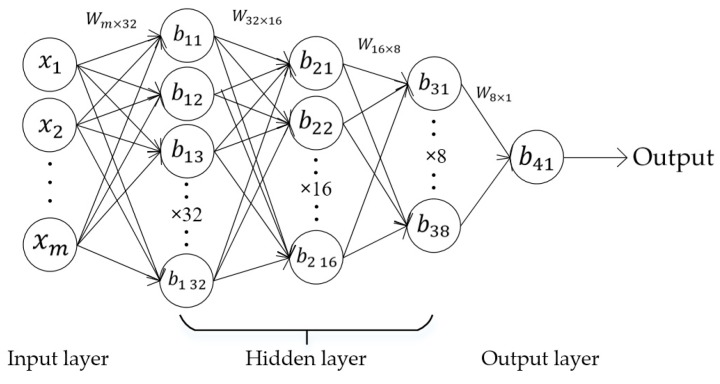
The structure of a BPNN-e.

The structure of a BPNN-e used for weight regression is depicted in Figure 10, containing an input layer, an output layer, and three hidden layers. Specifically, the number of neurons in the output layer was 1, and the number of neurons in the three hidden layers was 32, 16, and 8, respectively. It is worth noting that the dimension of the input of a BPNN-e varied with the number of vehicle axles. Let us take a six-axle vehicle as an example. The six-axle vehicle would generate six crests on the signal, so the inputs of the BPNN-e would include 2 × 6 areas of each crest, 2 × 5 distances between crests, and one vehicle speed (i.e., the dimension of the input is 23).

## 5. Implementation and Evaluation

**Figure 11:**
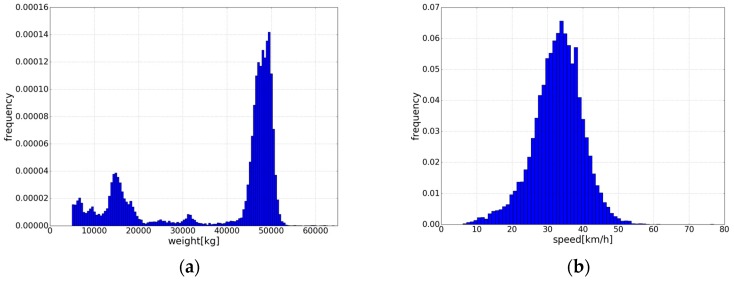
(**a**) The sample distribution over weights; (**b**) The sample distribution over speeds.

The MS-WIM system with the layout shown in Figure 1 was deployed on a public road. Meanwhile, a static weighing system was equipped on the same road so that the real weight of a vehicle was available. To evaluate our method, we used data collected from 28 August 2017 to 13 September 2017 as our dataset, which includes 324,408 samples generated by 5793 vehicles that passed. It is worth noting that samples collected by several failed sensors were deleted. As mentioned before, the average speed of a passing vehicle was computed by utilizing two loop sensors. The range of vehicle weight in our dataset is 5000–63,951 kg, and the speed range is 6.1–57.9 km/h. The distributions of all the samples over weight and speed are shown in Figure 11.

Furthermore, we implemented our method with the help of some tools. In particular, the short-time Fourier transform algorithm *stft* in *scipy* and the PCA algorithm in *scikit-learn* were used in our implementation. The width of the window function was set as 100, that is, each time 100 data points were selected as a small segment for Fourier transform. The dimension of the spectrum amplitude obtained by the short-time Fourier transform of the scaled signal was 12,597. The standard of the number of principal components to select was that the contribution of the cumulative variance of the principal component selected was up to 99%. After PCA dimensionality reduction, the dimension of spectrum amplitude was reduced from 12,597 to 8602 (i.e., the dimension decreased by 46.44%).

Both BPNN-i and BPNN-e were built on the top of *Tensorflow*. The number of neurons in the input layer was set to 8602 for BPNN-i and to 23 for BPNN-e. The weights of BPNN-i and BPNN-e were randomly initialized with random values satisfying a normal distribution, in which the mean was 0 and the variance was $\sqrt{2/n}$ (*n* is the number of the training data). All the bias values were initialized to zero. For the network configuration of BPNN-i, a small batch gradient descent method was used for training in which the batch size was 100 and the loss function was cross entropy. The activation function was sigmoid. For BPNN-e, the loss function was MSE (mean squared error), and Adaptive Moment Estimation (Adam) was chosen as the optimization algorithm. The activation function of BPNN-e is Rectified Linear Units (RELU). In addition, in order to prevent the neural network from overfitting, L2 regularization was added during the training for both BPNNs.

**Figure 12:**
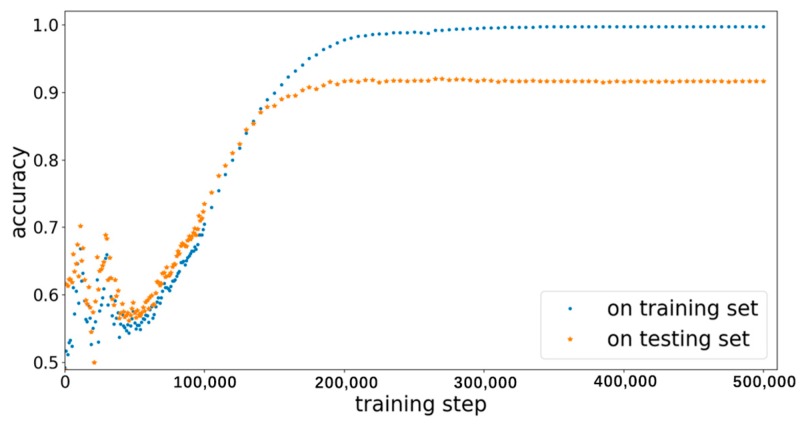
The changes of classification accuracy in the training and testing sets.

BPNN-i and BPNN-e were trained separately to achieve their respective goals. All experiments were carried out on a PC with i7-7500 and 8 G memory (Lenovo, Beijing, China). First, the BPNN-i was trained to select ideal samples. Eighty percent of samples in the dataset (i.e., 259,526 samples) were randomly selected as the training set; the remaining were used as the testing set. The samples in the dataset were manually labeled as ideal or not. The accuracy of identification was the ratio of the number of the samples with correct classification to the total number of the samples. The changes of identification accuracy in the training set and the testing set are shown in Figure 12. Eventually, the identification accuracy in the testing set was 92.04%.

To train and test the prediction network BPNN-e, 55,000 ideal samples were used, which were divided into a training set and a testing set according to a ratio of 4:1. To measure the accuracy of estimation in the testing set, the relative error defined by Equation (13) [9] was used:

(13) $$E=\frac{\left|W_{r}-W_{e}\right|}{W_{r}}\times 100\%,$$

where:

E is the relative error;
$W_{e}$ is the gross vehicle weight predicated by the trained BPNN-e; and
$W_{r}$ is the real vehicle weight computed by the static weighing system.

Overall, the relative error of 96.9% samples in the testing set was less than 5%. For each vehicle, the total time spent engaged in the data pre-process, identification, and predication was less than 3 s on average. We further investigated the relationship between weight, speed, and accuracy. The accuracies of estimation for different vehicle weights and speeds are shown in Table 1 and Table 2 respectively. As seen in Table 1, larger errors occurred at vehicle weights between 20,000–40,000 kg, while there were fewer training samples in this interval. However, as shown in Table 2, the average relative error had no obvious relationship with the number of training samples, and only slightly increased as the vehicle speed increased. The result indicates that more training data with different weights are needed to improve accuracy further.

## 6. Conclusions

In this paper, we presented a neural-network-based method to estimate gross vehicle weight for an MS-WIM system. The main contribution of this work is the integration of ideal sample identification into weight estimation. Experiments were conducted on particular populations of data obtained from real road traffic, and our results demonstrate the overall effectiveness of our method. For an MS-WIM system with sensors embedded under the pavement, the change of pavement characteristics will inevitably affect dynamic loads measured by sensors, which could subsequently increase estimation bias. To this end, both identification and predication networks need to be regularly retrained on new data so as to respond to the changes.

Like other learning-based methods, the accuracy performance of our method heavily relied on training data. Further evaluation will require more comprehensive data with larger variations in weight and speed. In addition, a new implementation that requires much less training data and transferring a trained network to a new MS-WIM system are also worthy of further study.

## Figures and Tables

**Table 1:** Average relative errors with different weights.

**Gross Vehicle Weight (t)**	10–20	20–30	30–40	40–50	50–60
**Number of Training Samples**	5850	447	595	35,004	2104
**Average Relative Error**	2.02%	4.68%	6.37%	1.19%	2.87%

**Table 2:** Average relative errors at different speeds.

**Speed (km/h)**	0–10	10–20	20–30	30–40	40–50	50–60
**Number of Training Samples**	54	1213	7858	23,932	10,225	718
**Average Relative Error**	1.68%	1.22%	1.45%	1.48%	1.47%	1.64%
